# Gene targeting using the *Agrobacterium tumefaciens*-mediated CRISPR-Cas system in rice

**DOI:** 10.1186/s12284-014-0005-6

**Published:** 2014-05-02

**Authors:** Rongfang Xu, Hao Li, Ruiying Qin, Lu Wang, Li Li, Pengcheng Wei, Jianbo Yang

**Affiliations:** 1 Key Laboratory of Rice Genetics Breeding of Anhui Province, Rice Research Institute, Anhui Academy of Agricultural Sciences, Hefei 230031, China; 2 Institute of Agricultural Engineering, Anhui Academy of Agricultural Sciences, Hefei 230031, China; 3 College of Life Sciences, Anhui University, Hefei 230031, China

**Keywords:** Gene targeting, CRISPR/Cas9, Rice, Agrobacterium, Stable transformation, Genome editing

## Abstract

**Background:**

The type II clustered, regularly interspaced, short palindromic repeat (CRISPR)/ CRISPR-associated protein 9 (Cas9) system is a novel molecular tool for site-specific genome modification. The CRISPR-Cas9 system was recently introduced into plants by transient or stable transformation.

**Findings:**

Here, we report gene targeting in rice via the *Agrobacterium tumefaciens*-mediated CRISPR-Cas9 system. Three 20-nt CRISPR RNAs were designed to pair with diverse sites followed by the protospacer adjacent motif (PAM) of the rice herbicide resistance gene *BEL*. After integrating the single-guide RNA (sgRNA) and Cas9 cassette in a single binary vector, transgenic rice plants harboring sgRNA:Cas9 were generated by *A. tumefaciens*-mediated stable transformation. By analyzing the targeting site on the genome of corresponding transgenic plants, the mutations were determined. The mutagenesis efficiency was varied from ~2% to ~16%. Furthermore, phenotypic analysis revealed that the biallelic mutated transgenic plant was sensitive to bentazon.

**Conclusions:**

Our results indicate that the agricultural trait could be purposely modified by sgRNA:Cas9-induced gene targeting. CRISPR-Cas9 system could be exploited as a powerful tool for trait improvements in crop breeding.

## Findings

Increases in the human population as well as in extreme weather events require the sustaining improvement of crop varieties. A directed, rapid, and low-cost method is critical for updating high-yield, multi-stress resistant varieties. Molecular marker-assisted selection (MAS) and genetic modification (GM) methods have advantages in capturing favorable agricultural traits. However, the targeted editing technology of key functional genes promises to be a powerful tool in accelerating varietal improvement. Zinc finger nucleases (ZFNs) and transcriptional activator-like effector nucleases (TALENs) have proven to be effective in plant-targeted genome editing (Chen and Gao [2013]; Li et al. [2012]; Shan et al. [2013a]; Zhang et al. [2013]); however, a simple, affordable, and high-throughput method is still needed. Recently, the bacterial type II clustered, regularly interspaced, short palindromic repeat (CRISPR)/CRISPR-associated protein (Cas) system has attracted attention due to its ability to induce sequence-specific genome editing. The site specificity is defined by the complementary base pairing of a small CRISPR RNA (crRNA). After annealing to a *trans*-activating crRNA (tracrRNA), the crRNA directly guides the Cas9 endonuclease to cleave the target DNA sequence. In humans, zebrafish, *Drosophila*, mice, and rats, genome editing can be achieved by simply combining an engineered *Streptococcus pyogenes Cas9* (*SpCas9*) and a synthetic single-guide RNA (sgRNA) consisting of the crRNA and the tracrRNA (Cong et al. [2013]; Feng et al. [2013]; Gratz et al. [2013]; Li et al. [2013a]; Wang et al. [2013]; Xiao et al. [2013]). The CRISPR/Cas9-induced genome editing was also recently performed in model plants (e.g., *Arabidopsis*, *Nicotiana benthamiana*) and crops (including wheat, rice and sorghum) by transient or stable transformation (Belhaj et al. [2013]; Feng et al. [2013]; Jiang et al. [2013]; Li et al. [2013b]; Mao et al. [2013]; Miao et al. [2013]; Nekrasov et al. [2013]; Shan et al. [2013b]; Xie and Yang [2013]). Here, we demonstrate that the CRISPR/Cas9 system could achieve efficient gene targeting in rice and modify the corresponding agricultural trait by *Agrobacterium tumefaciens*-mediated stable transformation.

**Figure 1:**
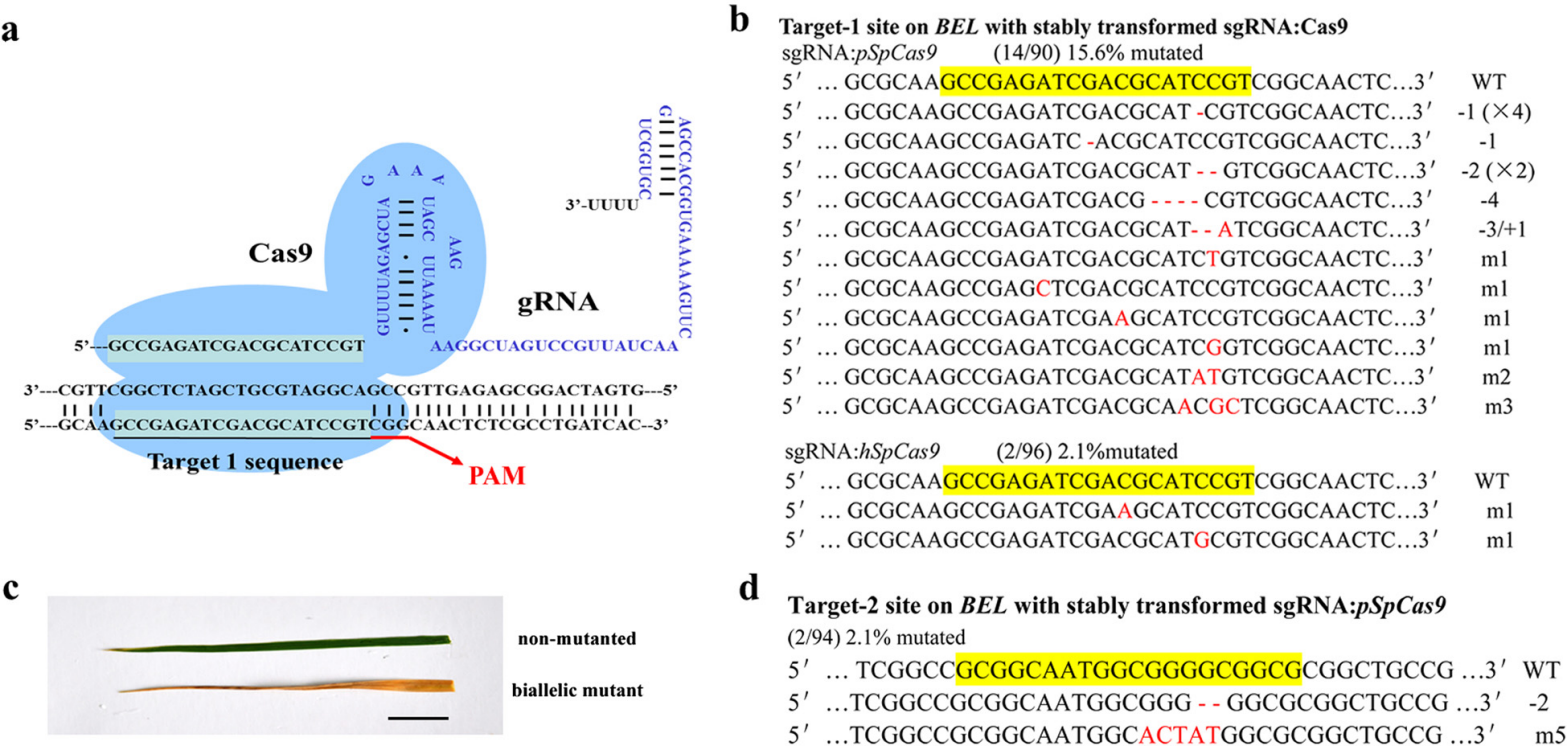
**Gene editing in rice using the stably expressed sgRNA:Cas9 system. (a)** Diagram of the sgRNA:Cas9 complex targeting the rice *BEL* gene at the target-1 site. PAM, the protospacer adjacent motif. gRNA, the guide RNA scaffold (Mali et al. [2013]). **(b)** sgRNA:Cas9-induced rice *BEL* gene mutations at the target-1 site. Upper: 14 mutants were identified in 90 independent lines transformed the target-1 sgRNA:*pSpCas9*. Lower: 2 mutants were identified in 96 independent lines transformed the target-1 sgRNA:*hSpCas9*. The yellow shadow marks the target sequence that is recognized by the crRNA. The deletions, insertions, and nucleotide substitutions are marked in red. **(c)** The phenotypes of the *bel* mutants. The leaves were sprayed with 1.5 g/L bentazon for 7 days. Upper: the transgenic plant without the target mutation; lower: the biallelic mutant with the site-specific mutation. Scale bar = 1 cm. **(d)** sgRNA:Cas9-induced rice *BEL* gene mutations at the target-2 site. 2 mutants were identified in 94 independent lines transformed the target-2 sgRNA:*pSpCas9*.

The rice *Bentazon Sensitive Lethal* (*BEL*, *LOC_Os03g0760200*) gene confers resistance to bentazon and sulfonylurea herbicides. The loss-of-function mutant *bel* is sensitive to the herbicides (Pan et al. [2006]). In two-line hybrid rice production, when male sterile lines are developed in the *bel* background, the problem of hybrid seed contamination by the selfing of sterile lines can be solved by simply spraying bentazon at the seedling stage. Although *BEL* has the potential to improve hybrid rice production safety, the limited natural genetic resources greatly restrict its application. Therefore, we selected *BEL* gene as the target for sgRNA:Cas9-based disruption. A 20-nucleotide (nt) region (target-1) at the 5’ end of a protospacer adjacent motif (PAM) in the second exon of *BEL* was selected as the editing target (Additional file 1: Method, Additional file 2: Figure S1). Complementary oligonucleotides were synthesized and were fused to the default sgRNA scaffold (Mali et al. [2013]) under control of an *Arabidopsis* U6-26 gene promoter (U6-26p). A double 35S promoter (pd35s) was used to drive a plant codon-optimized *SpCas9* (*pSpCas9*) coding region. In order to improve the efficiency of delivery, both of the sgRNA and Cas9 expression cassette were subcloned into a single binary vector (Figure 1a, Additional file 3: Figure S2). The construct was introduced into the embryonic calli of *japonica* rice cv. Nipponbare via *A. tumefaciens*, and independent transgenic events were isolated in the presence of hygromycin (Additional file 1: Method). The target region was amplified from the genomic DNA of transgenic plants and was Sanger-sequenced to detect sequence alterations. 15 mutations, including nucleotide deletions and substitutions, were identified in 14 lines from 90 independent transgenic plants (Figure 1b), indicating sgRNA:Cas9-induced gene targeting in rice.

To determine the effect of sgRNA:Cas9-directed gene targeting, we investigated the herbicide resistance of the mutated plants. A transgenic line with biallelic mutations (a 1-nt deletion of the 17th “C” and a 4-nt deletion of the 14th “CATC”, as shown in the first and the fourth mutated sequence in Figure 1b) was selected. The biallelic mutated plant and non-mutated transgenic lines were sprayed with bentazon. After 7-day incubation, the control leaves from the non-mutated transgenic plants remained green; however, the biallelic mutated leaves became severely wrinkled and yellowed (Figure 1c), confirming the functional disruption of the *BEL* gene.

To investigate influencing factors of the rice sgRNA:Cas9 mutagenesis efficiency, the gene targeting on 2 regions (a 20 nt region located in the first exon of *BEL* referred as the target-2 v.s. the target-1, Additional file 2: Figure S1) were performed using 2 codon types of Cas9 (humanized *SpCas9*, *hSpCas9* v.s. *pSpCas9*). After stably transformed by *Agrobacterium*, target regions of transgenic plants were screened. On the target-1, only 2 mutants were identified from 96 independent lines using *hSpCas9* (2.1% mutated), compared to the 15.6% mutagenesis frequency using *pSpCas9* (Figure 1b). The significant different on the mutagenesis frequency at the target-1 (P < 0.01, χ^2^ test) might be related to the higher level of Cas9 protein accumulation in the *pSpCas9* transgenic plants than in the *hSpCas9* transgenic plants (Additional file 3: Figure S2), similar to previous results that were obtained by transient assays (Li et al. [2013b]). Although sgRNA expression is considered as the limiting factor for effective mutagenesis (Li et al. [2013b]), our result suggests that the accumulation of Cas9 could also increase the targeting efficiency. The mutagenesis efficiencies were further determined on the target-2. For the target-2 sgRNA-*pSpCas9* construct, 94 independent transgenic lines were generated, and 2 mutants were identified (2.1% mutated). Meanwhile, transgenic plants harboring the target-2 sgRNA-*hSpCas9* were generated; however, no mutant was found among all of 96 transgenic lines (Figure 1d). Theoretically, every (N)_20_-NGG in genome could be recognized as the target site for sgRNA:Cas9-based gene targeting; however, the mutagenesis efficiency markedly varied between the 2 targeted sites using the same *pSpCas9* (P < 0.01, χ^2^ test). The target-2 was located in a GC-rich region (85% GC content at the target site and 71% within the 100-bp region comprising the target), whereas the GC content of the target-1 was relatively lower (65% at the target site and 60% within the 100-bp region comprising the target). In the absence of further statistical data, we speculate that the mutagenesis efficiency might be correlated with the nucleotide composition or the epigenetic state of target regions.

Several reports have suggested that the first nucleotide of the target sequence should be ‘G’, if the U6 promoter was used to drive sgRNA expression (Li et al. [2013b]; Mali et al. [2013]). This limitation would largely restrict potential mutation sites in genome editing. Therefore, we tested the stringency of the nucleotide on the first position. A 20-nt sequence beginning with ‘A’ was selected and was marked as the target-3 (Additional file 2: Figure S1). The gene targeting was performed with the binary vector containing *pSpCas9*. Among 96 independent transgenic lines, we identified 2 mutants (Additional file 4: Figure S3), suggesting that the ‘G’ is not absolutely required on the first position of the target sequence driven by the U6 promoter.

Since the specificity of sgRNA:Cas9 system became a major concern of the application (Hsu et al. [2013]), the off-target efficiency was further investigated. After searching rice genome using the target-1 sequence, three highly identical sites were found. Their sequences had 1-base or 3-base mismatches to the target-1 sequence. All of three genomic regions were examined in the target-1 sgRNA-*pSpCas9* transgenic lines. However, we did not observe any mutation at these sites by sequencing (Additional file 5: Table S1).

The delivery method of CRISRP/Cas system would be an important part of the potential application for crop improvement. The efficiency of gene targeting using the biolistic transformation method is relatively higher than the efficiency obtained in this study (Shan et al. [2013b]). Biolistic transformations frequently lead to multi-insertion copies insertion in transgenic plants, and possibly cause higher expression levels of sgRNA and Cas9 protein. However, the transgenes induced by *A. tumefaciens* usually exhibit a much higher single-site insertion frequency (Dai et al. [2001]), and the insertions should be easier to locate in the plant genome. Therefore, the genome would be easier to be identified in progenies of *A. tumefaciens*-mediated sgRNA:Cas9-targeting rice. In the near future, the direct application of *A. tumefaciens-*mediated sgRNA:Cas9-induce gene targeting on key functional genes would become a promising biotechnological strategy to facilitate the breeding of rice and other major crops which transgenic technologies are available.

## Accession codes

The accession number of *BEL* gene is NCBI GeneBank: DQ341412.

## Abbreviations

CRISPR: Clustered, regularly interspaced, short palindromic repeat: 

Cas: CRISPR-associated protein: 

PAM: Protospacer adjacent motif: 

sgRNA: Single-guide RNA: 

MAS: Molecular-assisted selection: 

GM: Genetic modification: 

ZFNs: Zinc finger nucleases: 

TALENs: Transcriptional activator-like effector nucleases: 

BEL: Bentazon Sensitive Lethal: 

Pd35s: double 35S promoter: 

U6-26p: *Arabidopsis* U6-26 gene promoter: 

crRNA: CRISPR RNA: 

tracrRNA: *trans*-activating crRNA: 

*SpCas9*: *Streptococcus pyogenes Cas9*: 

*hSpCas9*: Humanized S*pCas9*: 

*pSpCas9*: Plant codon-optimized *SpCas9*: 

## Competing interests

The authors declare no potential competing interests.

## Authors’ contributions

PW and JY conceived and designed the experiments. RX, HL, RQ, LW and LL performed the experiments and analysis. RX, HL, PW and JY discussed the data and wrote the paper. All authors read and approved the final manuscript.

## Additional files

## Supplementary Material

Additional file 1: Method: Plasmid construction and rice transformation. Click here for file

Additional file 2: Figure S1. Target loci in rice *BEL* gene. Click here for file

Additional file 3: Figure S2. Description of the sgRNA:Cas9 binary vector and the expression of Cas9. A, T-DNA insertion region of sgRNA:Cas9 binary vector. The *Arabidopsis* U6-26 gene promoter (U6-26p) and its terminator (U6 ter) were used to express sgRNA; The double 35S promoter and the NOS terminator were used to express 3XFLAG-tagged human or plant codon-optimized *spCas9*; The hygromycin was used as the plant selection marker for the vector. NLS, nuclear localization sequence. B, The plant codon-optimized *spCas9* showed a relatively higher expression level than human codon-optimized *spCas9* in transgenic plants by detecting FLAG-tag. The ACTIN were used as an internal control. Click here for file

Additional file 4: Figure S3. Site-specific mutations of transgenic plants at the target-3 with an ‘A’ at the start position of the 20 bp sequence. The yellow shadow marks the target sequence recognized by crRNA. The blue underline indicates the protospacer adjacent motif (PAM). DNA mutations are showed in red as letters and dashes, respectively. Click here for file

Additional file 5: Table S1. The detection on off-target mutations on the transgenic plants with the target-1 sgRNA:*pSpCas9*. **Table S2.** List of PCR primers used and their application. Click here for file
