# Supplementary material for: Gene targeting using the Agrobacterium tumefaciens-mediated CRISPR-Cas system in rice
Source: Rice (N Y). 2014 May 2;7(1):5. doi: 10.1186/s12284-014-0005-6 (PMC4052633; doi:10.1186/s12284-014-0005-6)
Supplement: Additional file 1: — Method: Plasmid construction and rice transformation. [file s12284-014-0005-6-S1.doc]

**Methods**

**Plasmid construction and rice transformation**.

For constructing the sgRNA cassette, the sequence of *Arabidopsis* U6-26 gene promoter (U6-26p) and terminator (U6 ter) were selected. A pre-described gRNA scaffold sequence was inserted between the promoter and the terminator. Two oppositely oriented *BsaHI* sites were placed between the promoter and sgRNA scaffold. The whole cassette was synthesized (Genewiz, China). For constructing the Cas9 cassette, a double 35S promoter (pd35S) and the terminator of the nopaline synthase gene (tNOS) were cloned. The coding region of *spCas9* were codon optimized for expression in human cell or plant (synthesized by Genewiz). The coding regions of Cas9 were fused in frame with a nuclear localization sequence (NLS) following a 3X*FLAG* tag at the 3’ end and inserted into *NotI*-*SacI* sites between pd35S and tNOS. For construction of the binary vector containing both of sgRNA and Cas9, a pGREEN vector 0179 was modified. The Cas9 expression cassette was inserted into *HindIII*-*EcoRI* sites on upstream of the *HPT* cassette. The sgRNA cassette was then inserted into *HindIII* site and plasmids with the 5’-3’ orientation fragment were selected for further construction.

For constructing gene targeting vectors, the synthesized target sequences listed in Supplementary Table 2 were annealed and inserted into the *BsaHI*-predigested binary vector. After selecting by kanamycin, the clones were verified by PCR using the forward primer located on U6-26p and the reverse complementary target sequences. After sequencing on target sites, the binary vectors transformed into the *Agrobacterium* *tumefaciens* EHA 105 strain containing the pSOUP vector . Calli of Japonica Nipponbare were infected, and hygromycin-resistant plants were regenerated as previously described .

**Mutation identification**

Genomic DNA (gDNA) was isolated from the mature leaves of the 8-weeks transgenic lines using a DNeasy Plant Kit (Qiagen, German). The fragments containing the target site were amplified from individual samples by site-specific primers that are listed in Supplementary Table 2 with the high-fidelity DNA polymerase *Phusion* (New England Biolabs, USA), where 20 ng leaf gDNA was used as PCR template in a 50 μl PCR reaction system for 30 amplification cycles. The fragments with expected size were purified from agarose gel, and the products were directly Sanger-sequenced by the corresponding forward primer. The samples that had abnormal chromatogram or sequence mutation were further sub-cloned into the pEASY-T vector (Transgene, China). The resultant transformants were identified by colony PCR. For each genomic DNA sample, at least six PCR-positive colonies were randomly selected and sequenced using a M13 primer. The mutations were identified by aligning to the reference sequences. The plant had two mutation types and 100% mutated clonal amplicons was considered as the biallelic mutant.

**Herbicide treatment**

The bentazon solution was diluted to 1.5 g/L by 1/2 liquid Murashige and Skoog medium (MS) media containing 0.1% Tween-20. The leaves of the mutant and control plants were sprayed for 3 days.

**Supplementary References**

Cong L, Ran FA, Cox D, Lin S, Barretto R, Habib N, Hsu PD, Wu X, Jiang W, Marraffini LA, Zhang F (2013) Multiplex Genome Engineering Using CRISPR/Cas Systems. Science 339 (6121):819-823. doi:10.1126/science.1231143

Duan Y, Zhai C, Li H, Li J, Mei W, Gui H, Ni D, Song F, Li L, Zhang W, Yang J (2012) An efficient and high-throughput protocol for Agrobacterium-mediated transformation based on phosphomannose isomerase positive selection in Japonica rice (Oryza sativa L.). Plant cell reports 31 (9):1611-1624. doi:10.1007/s00299-012-1275-3

Hellens R, Edwards EA, Leyland N, Bean S, Mullineaux P (2000) pGreen: a versatile and flexible binary Ti vector for Agrobacterium-mediated plant transformation. Plant Mol Biol 42 (6):819-832. doi:10.1023/a:1006496308160

Mali P, Yang L, Esvelt KM, Aach J, Guell M, DiCarlo JE, Norville JE, Church GM (2013) RNA-Guided Human Genome Engineering via Cas9. Science 339 (6121):823-826. doi:10.1126/science.1232033
