# Supplementary material for: Gene targeting using the Agrobacterium tumefaciens-mediated CRISPR-Cas system in rice
Source: Rice (N Y). 2014 May 2;7(1):5. doi: 10.1186/s12284-014-0005-6 (PMC4052633; doi:10.1186/s12284-014-0005-6)
Supplement: Additional file 2: Figure S1. — Target loci in rice BEL gene. [file s12284-014-0005-6-S2.doc]

**Figure S1.** Target loci in rice *BEL* gene.


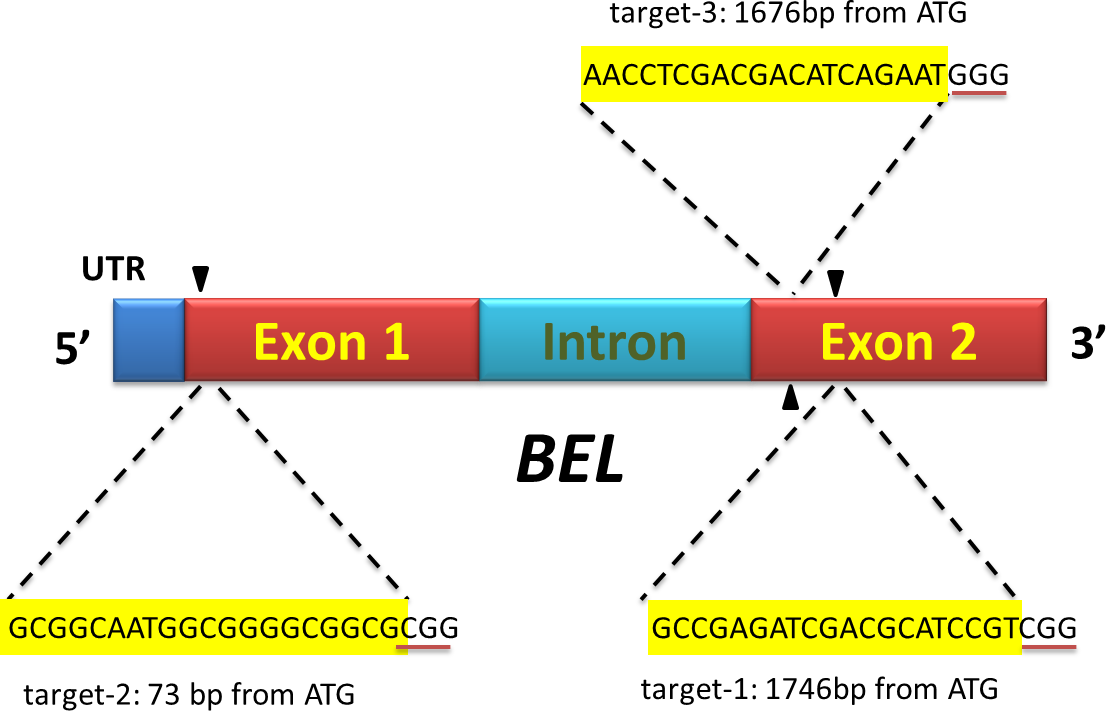


The target sites were marked by yellow shadow and the following PAM were underlined by red. The position of the first nucleotide of each target was labeled.
