# Supplementary material for: Gene targeting using the Agrobacterium tumefaciens-mediated CRISPR-Cas system in rice
Source: Rice (N Y). 2014 May 2;7(1):5. doi: 10.1186/s12284-014-0005-6 (PMC4052633; doi:10.1186/s12284-014-0005-6)
Supplement: Additional file 3: Figure S2. — Description of the sgRNA:Cas9 binary vector and the expression of Cas9. A, T-DNA insertion region of sgRNA:Cas9 binary vector. The Arabidopsis U6-26 gene promoter (U6-26p) and its terminator (U6 ter) were used to express sgRNA; The double 35S promoter and the NOS terminator were used to express 3XFLAG-tagged human or plant codon-optimized spCas9; The hygromycin was used as the plant selection marker for the vector. NLS, nuclear localization sequence. B, The plant codon-optimized spCas9 showed a relatively higher expression level than human codon-optimized spCas9 in transgenic plants by detecting FLAG-tag. The ACTIN were used as an internal control. [file s12284-014-0005-6-S3.doc]

**Figure S2.**

**
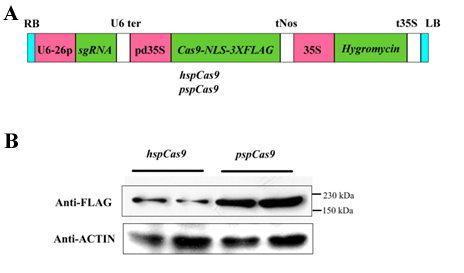
**

**Figure S2.** Description of the sgRNA:Cas9 binary vector and the expression of Cas9. A, T-DNA insertion region of sgRNA:Cas9 binary vector. The *Arabidopsis* U6-26 gene promoter (U6-26p) and its terminator (U6 ter) were used to express sgRNA; The double 35S promoter and the NOS terminator were used to express 3XFLAG-tagged human or plant codon-optimized *spCas9*; The hygromycin was used as the plant selection marker for the vector. NLS, nuclear localization sequence. B, The plant codon-optimized *spCas9* showed a relatively higher expression level than human codon-optimized *spCas9* in transgenic plants by detecting FLAG-tag. The ACTIN were used as an internal control.
