# Supplementary material for: Gene targeting using the Agrobacterium tumefaciens-mediated CRISPR-Cas system in rice
Source: Rice (N Y). 2014 May 2;7(1):5. doi: 10.1186/s12284-014-0005-6 (PMC4052633; doi:10.1186/s12284-014-0005-6)
Supplement: Additional file 4: Figure S3. — Site-specific mutations of transgenic plants at the target-3 with an ‘A’ at the start position of the 20 bp sequence. The yellow shadow marks the target sequence recognized by crRNA. The blue underline indicates the protospacer adjacent motif (PAM). DNA mutations are showed in red as letters and dashes, respectively. [file s12284-014-0005-6-S4.doc]

**Figure S3.**

**
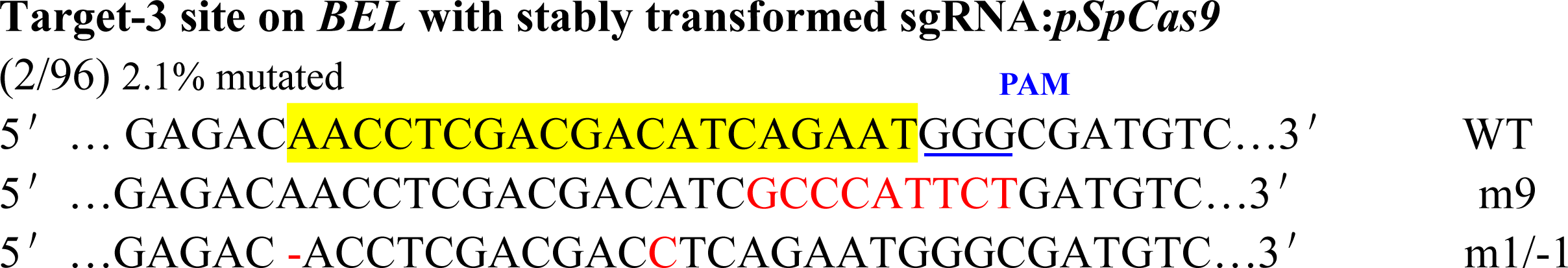
**

**Figure S5.** Site-specific mutations of transgenic plants at the target-3 with an ‘A’ at the start position of the 20bp sequence. The yellow shadow marks the target sequence recognized by crRNA. The blue underline indicates the protospacer adjacent motif (PAM). DNA mutations are showed in red as letters and dashes, respectively.
