# Supplementary material for: Gene targeting using the Agrobacterium tumefaciens-mediated CRISPR-Cas system in rice
Source: Rice (N Y). 2014 May 2;7(1):5. doi: 10.1186/s12284-014-0005-6 (PMC4052633; doi:10.1186/s12284-014-0005-6)
Supplement: Additional file 5: Table S1. — The detection on off-target mutations on the transgenic plants with the target-1 sgRNA:pSpCas9. Table S2. List of PCR primers used and their application. [file s12284-014-0005-6-S5.doc]

**Table S1,** The detection on off-target mutations on the transgenic plants with the target-1 sgRNA:*pSpCas9*;

The mismatching bases are marked by red shadow

| Off-target Location | Sequence of the off-target site | Mismatching number | Mutated events |
| --- | --- | --- | --- |
| Chromosome 3 | GCCGAGATCGACGCGTCCGT | 1 | 0/88 |
| Chromosome 12 | ACCGAGATGTACGCATCCGT | 3 | 0/90 |
| Chromosome 10 | GCCGAGG-TCGACGCATCCCC | 3 | 0/82 |

**Table S2.** List of PCR primers used and their application

| Primer name | Sequence (5’-3’) | Application |
| --- | --- | --- |
| BEL KO1 P1  BEL KO1 P2 | ATTGCCGAGATCGACGCATCCGT  AAACACGGATGCGTCGATCTCGG | Generating sgRNA for the target-1 |
| BEL KO2 P1  BEL KO2 P2 | ATTGCGGCAATGGCGGGGCGGCG  AAACCGCCGCCCCGCCATTGCCG | Generating sgRNA for the target-2 |
| BEL KO3 P1  BEL KO3 P2 | ATTGACCTCGACGACATCAGAAT  AAACATTCTGATGTCGTCGAGGT | Generating sgRNA for the target-3 |
| BEL1/3-gDNA-F  BEL1/3-gDNA-R | AATCTTAGTTCCACCCTCTTGC  ACATGAGTTCGTCCTGGAGATG | Screen mutation on the target-1and the target-3 |
| BEL2-gDNA-F  BEL2-gDNA-R | AACGCCTACATTATTGCCATTC  CGACGACCTGCTTAAACTCCTG | Screen mutation on the target-2 |
